# ASO Author Reflections: Surgery for pT3 and pT4 Cutaneous Squamous Cell Carcinomas of the Head and Neck Provides Robust Outcomes Against Which Emerging Treatment Modalities Should be Compared to Determine Their Role in the Standard of Care

**DOI:** 10.1245/s10434-022-11696-w

**Published:** 2022-04-07

**Authors:** Amanda E. Yung, Sydney Ch’ng

**Affiliations:** 1 Sydney Medical School, University of Sydney, Sydney, NSW Australia; 2 Royal Prince Alfred Hospital Institute of Academic Surgery, Sydney, NSW Australia; 3 Faculty of Medicine and Health, The University of Sydney, Sydney, NSW Australia; 4 Melanoma Institute Australia, University of Sydney, Camperdown, NSW Australia; 5 Department of Plastic and Reconstructive Surgery, Royal Prince Alfred Hospital, Sydney, NSW Australia

## Past

The current standard of care for pT3/4 cutaneous squamous cell carcinomas of the head and neck (HNcSCC) consists of surgery and post-operative radiotherapy. However, a number of patients may be considered incurable with surgery due to the presence of multiple-recurrent disease or inability to achieve clear microscopic margins. In the past, alternatives to surgery for advanced HNcSCC, such as definitive radiotherapy or chemotherapy, have been much inferior to surgery and were associated with significant adverse events.^1^

## Present

Based on encouraging data from recent clinical trials of immune checkpoint inhibitors (ICI), cemiplimab and pembrolizumab have been granted FDA approval for use in locally advanced and metastatic HNcSCC not curative with surgery or radiotherapy.^2,3^ To allow better integration of conventional and emerging treatment modalities, we reviewed the surgical treatment outcomes of our patient cohort with pT3/4 HNcSCC.^4^ Our real-world experience demonstrated a 5-year locoregional control rate of 62.0%, disease-specific survival rate of 83.7%, and overall survival rate of 71.9%. LRC was reduced in the presence of margin involvement and previous treatment (radiotherapy/surgery). These adverse features should guide the use of adjuvant or alternative therapy.

## Future

This study is to date the largest dataset of advanced HNcSCC patients treated with standard of care of surgery with/without postoperative radiotherapy/medical therapy with long-term follow-up before wider use of ICI immunotherapy. It provides a benchmark against which potential treatment alternatives may be compared. While currently reported outcomes of ICI as primary or adjuvant treatment for advanced HNcSCC are promising, long-term clinical trial data and real-world outcomes are still emerging. Further studies assessing interactions between preoperative functional status and surgical outcomes/complications, and prediction of individual response to ICI immunotherapy are required to allow better selection of patients most suitable for surgery versus alternative primary or (neo)adjuvant therapies for advanced HNcSCC.
